# Novel Therapeutic Strategy for Renal Cell Carcinoma: Niclosamide Enhances Sunitinib Efficacy via DNA Repair and Cell Cycle Pathways

**DOI:** 10.3390/ijms262210922

**Published:** 2025-11-11

**Authors:** Ae Ryang Jung, Ga Eun Kim, Mee Young Kim, Seung Ah Rhew, Dongho Shin, U-Syn Ha, Sung-Hoo Hong, Ji Youl Lee, Sae Woong Kim, Yong Hyun Park

**Affiliations:** Department of Urology, Seoul St. Mary’s Hospital, College of Medicine, The Catholic University of Korea, Seoul 06591, Republic of Korea; exactly@nate.com (A.R.J.); silscm@nate.com (G.E.K.); iris1774@hanmail.net (M.Y.K.); sarhew@naver.com (S.A.R.); eds8813@naver.com (D.S.); ushamd@catholic.ac.kr (U.-S.H.); toomey@catholic.ac.kr (S.-H.H.); uroljy@catholic.ac.kr (J.Y.L.); ksw1227@catholic.ac.kr (S.W.K.)

**Keywords:** renal cell carcinoma, sunitinib, niclosamide, synergistic effect, DNA repair, cell cycle

## Abstract

Tyrosine kinase inhibitors (TKIs), such as sunitinib and sorafenib, are standard treatments for renal cell carcinoma (RCC). However, most patients treated with these drugs eventually develop drug resistance and relapse; therefore, new treatment options for RCC are urgently required. Recent studies have focused on combination therapies targeting distinct molecular pathways that may produce synergistic effects and help overcome drug resistance in RCC. Niclosamide, an anthelmintic agent, is effective against various cancers; however, its potential in combination with sunitinib for treating RCC has not been evaluated. In this study, we assessed the therapeutic efficacy of niclosamide in combination with sunitinib against RCC and explored the underlying molecular mechanisms. Niclosamide alone inhibited RCC cell proliferation, whereas its combination with sunitinib produced a synergistic anticancer effect, both in vitro and in vivo. RNA sequencing (RNA-seq) and bioinformatic analyses showed that niclosamide modulated critical pathways, including *BRIP1*- and *FANCA*-mediated DNA repair and *E2F2*-regulated cell cycle progression. These findings provide proof-of-concept that niclosamide enhances TKI efficacy through modulation of DNA repair and cell cycle pathways, supporting the rationale for DNA damage response (DDR)-targeted combination strategies in RCC.

## 1. Introduction

Renal cell carcinoma (RCC) is one of the most common malignancies worldwide, and its incidence is steadily increasing [1]. RCC comprises various histological subtypes; the most frequent subtypes are clear cell RCC (ccRCC), papillary RCC (pRCC), and chromophobe RCC (chRCC). Other subtypes are rare, each accounting for approximately 1% of RCC cases [2].

Tyrosine kinase inhibitors (TKIs), such as sunitinib and sorafenib, have been approved to treat advanced RCC [3]. Sunitinib inhibits several receptor tyrosine kinases, including the vascular endothelial growth factor receptor (VEGFR), platelet-derived growth factor receptor (PDGFR), Fms-related tyrosine kinase 3 and c-KIT, and is widely used as a first-line therapy [3,4]. Despite its therapeutic effects, most patients receiving sunitinib develop resistance within 6–15 months. Acquired resistance leads to disease relapse and remains an important clinical challenge [5,6].

To overcome this limitation, new therapeutic strategies, particularly combination approaches that target complementary pathways, are required [7]. Among these, drug repositioning has emerged as a promising approach, as it is cost-effective and time-efficient. In particular, combining repurposed agents with established treatments may yield synergistic effects and help overcome therapeutic resistance in cancer [8].

Niclosamide, an FDA-approved anthelminthic drug, has pleiotropic properties including anti-inflammatory, bronchodilatory, antibacterial, and anticancer activities in various tumor types. Unlike conventional TKIs, niclosamide simultaneously targets multiple oncogenic pathways, including the inhibition of Wnt/β-catenin, mTOR, and STAT3 signaling [9]. These pathways are critically involved in RCC pathogenesis and progression [10]. In addition, niclosamide induces DNA damage in prostate cancer cells by suppressing FOXM1, which mediates DNA damage response (DDR) [11], suggesting a distinct and multifaceted mechanism of action. The ability of niclosamide to disrupt several cancer-related pathways provides a strong rationale for its use in combination with other targeted therapies, such as TKIs, to more effectively impair RCC cell survival.

Notably, niclosamide exhibits anticancer activity against RCC and enhances the therapeutic effects of sorafenib [12,13]. However, the potential role of niclosamide in RCC remains unclear. The effects of combining niclosamide with sunitinib have not yet been studied. Considering the distinct mechanisms of action of these two agents, we hypothesized that their combination would synergistically inhibit RCC growth.

In this study, we aimed to assess the therapeutic efficacy of the niclosamide–sunitinib combination and elucidate its underlying mechanisms, with particular emphasis on DDR and cell cycle regulation.

## 2. Results

### 2.1. Niclosamide and Sunitinib Individually Exhibit Anticancer Effect in RCC

**Figure 1:**
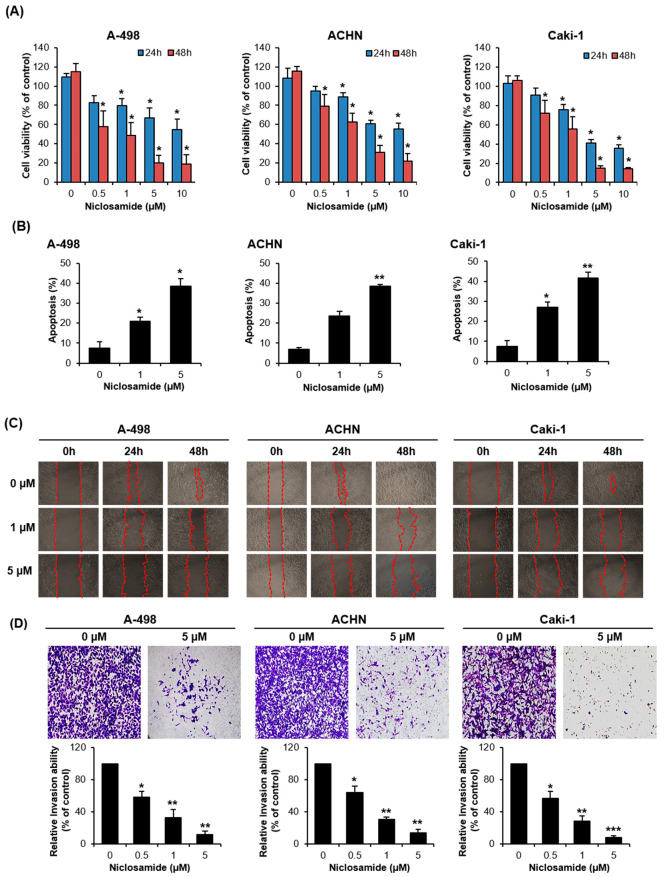
Anticancer effect of niclosamide in renal cell carcinoma (RCC). (**A**) A-498, ACHN, and Caki-1 cells were treated with the indicated concentrations of niclosamide for 24 and 48 h. Cell viability was measured by WST assay. Bar graphs represent the mean ± standard deviation (SD; * *p* < 0.05 compared to control at the same time point). (**B**) The cells were treated with the indicated niclosamide concentrations for 48 h. Apoptotic cell populations were quantified by flow cytometry following Annexin V-FITC/PI staining and are presented as bar graphs. Data are expressed as mean ± standard deviation (SD; * *p* < 0.05, ** *p* < 0.01 compared to control). (**C**) Cell migration was assessed by wound healing assays following niclosamide treatment and visualized by microscopy (magnification, ×40). Red dashed lines indicate the wound margins at each time point. Images for each treatment group (0/24/48 h) were obtained from the same independent repeat, well, and field of view; n = 3 independent experiments (**D**) Cell invasion was evaluated using Matrigel-coated Transwell assays after treatment with 0.5, 1, 5 μM niclosamide for 48 h. Invaded cells were imaged by microscopy (×100). Data are expressed as mean ± SD (* *p* < 0.05, ** *p* < 0.01, *** *p* < 0.001 as compared to control).

To evaluate the individual anticancer efficacy of each drug, RCC cell lines were treated with niclosamide and sunitinib. As expected, sunitinib dose-dependently inhibited RCC cell viability (Supplementary Figure S1). Niclosamide, an FDA-approved anthelmintic drug, significantly reduced RCC cell line viability dose- and time-dependently (Figure 1A). Niclosamide significantly increased the apoptotic cell population dose-dependently in all three RCC cell lines (Figure 1B and Supplementary Figure S2). Moreover, niclosamide markedly suppressed cell migration and invasion dose-dependently (Figure 1C and D). These results indicate that niclosamide possesses potent anticancer properties against RCC.

### 2.2. Combination of Sunitinib and Niclosamide Exerts Synergistic Anticancer Effects In Vitro

**Figure 2:**
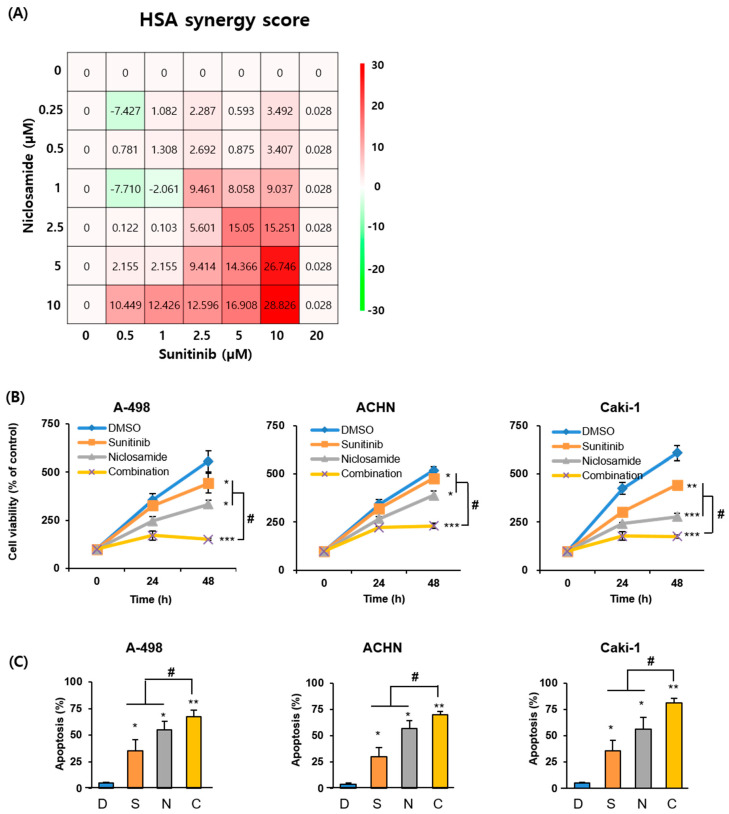
Synergistic effects of the combination of sunitinib and niclosamide. (**A**) ACHN cells were treated with various concentrations of sunitinib and niclosamide for 48 h, and cell viability was measured. Synergy was analyzed using SynergyFinder 2.0 based on the Highest Single Agent (HSA) model. The synergy landscape displays numerical synergy scores and corresponding color gradients: red indicates synergy (score > 0), green indicates antagonism (scores < 0), and white represents additive effects (score = 0). Each synergy score represents the interaction at a specific concentration pair. (**B**) A-498, ACHN, and Caki-1 cells were treated with 2.5 µM sunitinib, 1 µM niclosamide, or their combination. Cell viability was measured by WST assay at the indicated time points. Bar graphs represent mean ± SD. (**C**) Cells were treated with 2.5 µM sunitinib, 1 µM niclosamide, or their combination, for 48 h. Apoptotic cell populations were quantified by flow cytometry following Annexin V-FITC/PI staining. Quantitative data are presented as mean ± SD. * *p* < 0.05, ** *p* < 0.01, *** *p* < 0.001 compared to control; # *p* < 0.01 compared to monotherapy. D, DMSO; S, sunitinib; N, niclosamide; C, combination.

Next, we investigated whether the combination of sunitinib and niclosamide exerted synergistic anticancer effects on RCC. ACHN cells were treated with various concentrations of both drugs for 48 h, and cell viability was measured. Synergy analysis was conducted using the Highest Single Agent (HSA) model in SynergyFinder 2.0 [14], based on data from seven different concentrations of each drug and their combination, with at least three replicates per condition (Supplementary Figure S3A–C). This combination exhibited a positive synergy score of 5.663, with the highest synergy (score: 28.826) in ACHN cells (Figure 2A), indicating a synergistic interaction. Notably, 2.5 µM sunitinib and 1 µM niclosamide combination demonstrated a synergy score of 9.46 with minimal drug concentrations (Figure 2A). Based on these results, the selected combination ratio was used in subsequent experiments. This combination therapy significantly reduced A-498, ACHN, and Caki-1 cell viability (Figure 2B) and increased apoptosis compared with either agent alone (Figure 2C and Supplementary Figure S4).

### 2.3. Combination Therapy Significantly Suppresses Tumor Growth In Vivo

To validate these findings *in vivo*, we established an ACHN xenograft model in nude mice. Tumor-bearing mice were treated daily with DMSO (vehicle), sunitinib, niclosamide or a combination of both. No significant toxicity or weight loss was observed during treatment.

**Figure 3:**
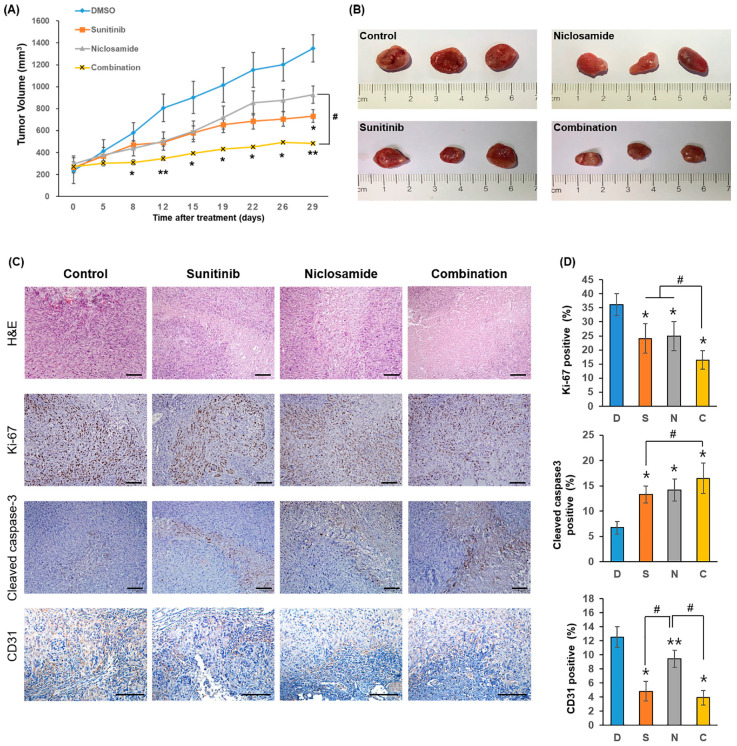
In vivo antitumor efficacy of the combination of sunitinib and niclosamide. (**A**,**B**) ACHN xenograft-bearing mice were treated with vehicle (1% DMSO and 1% Tween-80 in PBS), sunitinib (20 mg/kg), niclosamide (20 mg/kg), or their combination (20 mg/kg each) for 29 days. Tumor volumes and body weights were measured twice weekly and are shown as mean ± standard error of the mean (SEM; n = 5 per group). Statistical comparisons were performed using two-way analysis of variance (ANOVA) followed by Tukey’s multiple comparisons test. * *p* < 0.05, ** *p* < 0.01 as compared to control group. # *p* < 0.01 as compared to monotherapy. (**C**,**D**) Tumors were harvested and analyzed by hematoxylin and eosin (H&E) and immunohistochemistry (IHC) for Ki-67, cleaved caspase-3, and CD31 (brown staining; Scale bar, 100 µm). Quantification of positive cells is shown as mean ± SD (* *p* < 0.05, ** *p* < 0.05 compared to control. # *p* < 0.05 compared to monotherapy). D, DMSO; S, sunitinib; N, niclosamide; C, combination.

Tumor growth in the DMSO group increased rapidly during the study period (Figure 3A). Both sunitinib and niclosamide monotherapy moderately suppressed tumor growth compared with the control. Consistently, final tumor weights and representative images also supported these findings (Figure 3B). At the study endpoint (day 29), the sunitinib group was significantly different from control (*p* = 0.0302), whereas the niclosamide group did not show any statistical significance (*p* = 0.1217). No significant difference was observed between the sunitinib and niclosamide groups (*p* = 0.2258).

Notably, the combination treatment markedly inhibited tumor growth than the control group beginning on day 8 (adjusted *p* = 0.0493), with differences becoming more pronounced on days 12, 15, 19, 22, 26, and 29 (all adjusted *p* < 0.05). In addition, a significant difference between the niclosamide and combination groups emerged from day 19 onward (*p* < 0.05). On day 29, tumors in the combination group were significantly smaller, showing a 2.79-fold reduction in volume relative to the control (*p* = 0.0119), a 1.91-fold reduction relative to the niclosamide group (*p* = 0.0040), and a 1.51-fold reduction relative to the sunitinib group, although the difference was not statistically significant (*p* = 0.0872).

Immunohistochemical analysis of tumor sections further supported the enhanced antitumor effect of the combination therapy. In the combination group, the expression of Ki-67, a proliferation marker, was markedly reduced, whereas that of cleaved caspase-3, an apoptotic marker, was significantly higher than in the control and monotherapy groups (Figure 3C and D). These findings underscore the synergistic antitumor effects achieved by the combined use of sunitinib and niclosamide, surpassing the benefits conferred by either agent alone.

To assess the anti-angiogenic effects, CD31 expression was evaluated. All treatment groups showed reduced CD31 expression relative to the control group, with the most pronounced reduction observed in the combination group. Interestingly, CD31 expression in the niclosamide group was significantly higher than that in the sunitinib and combination groups, but still lower than that in the control group. Quantitative analysis of CD31-positive vessel area showed that micro-vessel density was lowest in the combination group, indicating an additive reduction in tumor vascularity compared with sunitinib alone. These data suggest that niclosamide may partially contribute to anti-angiogenic effects while exerting additional antitumor activity through distinct mechanisms. Taken together, these results indicate that the enhanced efficacy of combination therapy results from the anti-angiogenic effects of sunitinib and complementary pathways modulated by niclosamide.

### 2.4. Niclosamide Induces Transcriptomic Alterations in RCC

**Figure 4:**
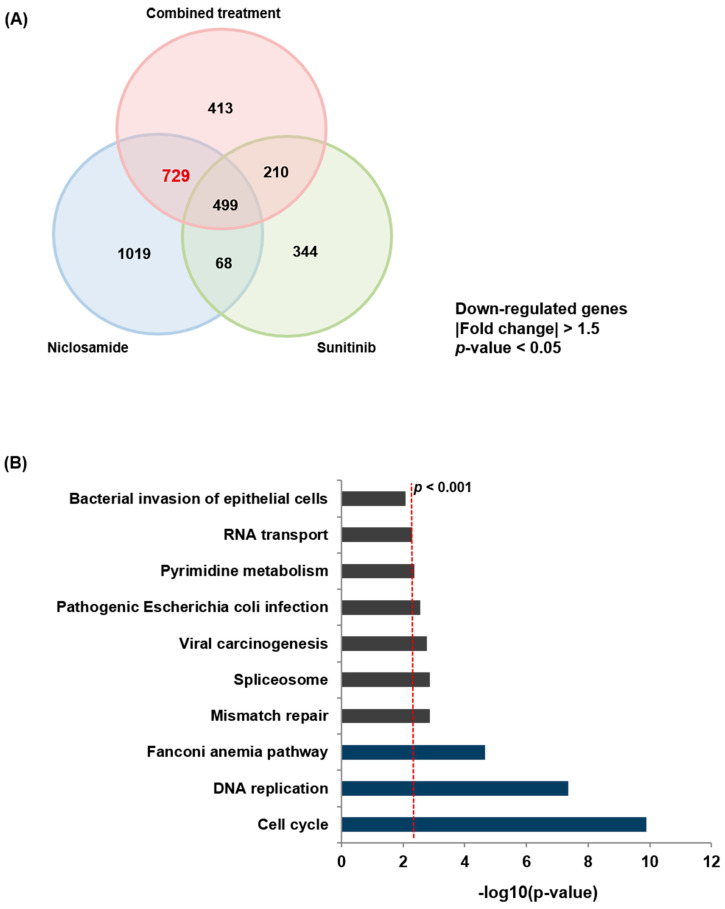
Identification of down-regulated genes and pathways affected by niclosamide. (**A**) RNA-seq analysis was performed on ACHN cells treated with 2.5 µM sunitinib and 1 µM niclosamide, or their combination, for 48 h. A Venn diagram illustrates the number of down-regulated genes in each treatment group (|fold change| > 1.5, *p* < 0.05). A total of 729 genes commonly down-regulated by both niclosamide and the combination are highlighted in red. (**B**) KEGG pathway analysis was conducted on the down-regulated genes identified in (**A**).

To elucidate the molecular mechanisms underlying niclosamide activity, RNA-seq was performed after treatment with sunitinib, niclosamide, or their combination, each compared with vehicle-treated controls. Differential expression analysis revealed that all three treatments significantly altered gene expression profiles. To identify niclosamide-driven molecular changes, we focused on genes commonly down-regulated by niclosamide alone and by the combination, but not by sunitinib (Figure 4A). A total of 729 genes were significantly down-regulated (|fold change| > 1.5, *p* < 0.05). KEGG pathway enrichment analysis of these niclosamide-associated genes (*p* < 0.001; Figure 4B and Supplementary Table S1) revealed significant enrichment in pathways related to the cell cycle (gene count: 24), DNA replication (gene count: 12), and Fanconi anemia (FA) pathway (gene count: 11). These results suggest that the major anticancer mechanisms of niclosamide involve disruption of cell cycle and DNA repair processes, thereby contributing to the synergistic activity observed with sunitinib.

### 2.5. Niclosamide Induces Go/G1 Arrest and Activates DDR

**Figure 5:**
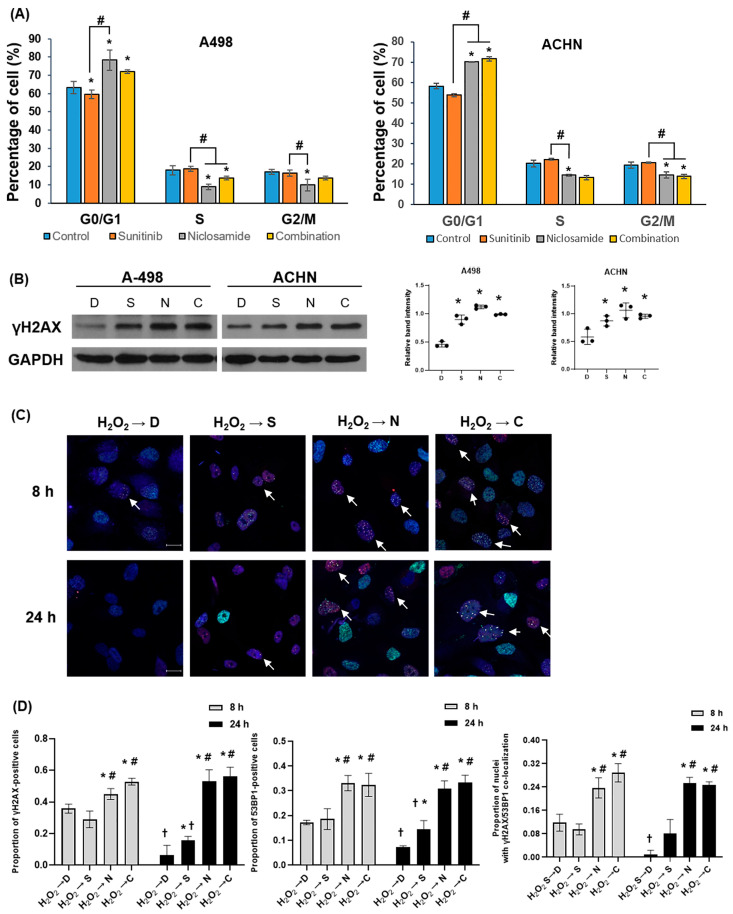
Improved anti-cancer efficacy of combination treatment via DNA damage and cell cycle arrest. (**A**) Cell cycle distribution was analyzed by flow cytometry, showing the percentages of cells in G0/G1, S, and G2/M phases. Data are expressed as mean ± SD (* *p* < 0.05 compared to the control group; # *p* < 0.05 compared to the sunitinib group). (**B**) γ-H2AX protein expression was detected by Western blot analysis; GAPDH served as a loading control. Representative blots from n = 3 independent experiments are shown. Densitometric quantification of γ-H2AX relative to GAPDH is presented as mean ± SD (n = 3). Statistical significance was determined by one-way ANOVA with post hoc tests. (* *p* < 0.05 compared to control). (**C**) Nuclear γ-H2AX (green) and 53BP1 (red) foci in A498 were visualized by immunofluorescence, with DAPI counterstaining (blue). γH2AX and 53BP1 Co-localization within the same nucleus is shown in merged images. White arrows indicated representative co-localized foci. Scale bar = 20 µm. (**D**) Proportions of γH2AX-, 53BP1-positive, and co-localized nuclei were quantified from multiple fields using ZEN lite software, version 3.12 (Carl Zeiss, Germany). Data are expressed as mean ± SD (* *p* < 0.05 compared to the H_2_O_2_ group at the same time point; # *p* < 0.05 compared to the sunitinib group at the same time point; † *p* < 0.05 between 8 h and 24 h within the same treatment). D, DMSO; S, sunitinib; N, niclosamide; C, combination.

To validate the transcriptomic findings, we observed the effects of niclosamide on cell cycle progression and DDR in RCC (Figure 5). Niclosamide, alone or in combination with sunitinib, significantly increased G0/G1 phase arrest in both A498 and ACHN cells (Figure 5A and Supplementary Figure S5). Specifically, 78% and 72% of A498 cells, and 70% and 71% of ACHN cells were arrested in G0/G1 phase following treatment with niclosamide alone or in combination, respectively.

Western blotting analyses revealed upregulation of γ-H2AX expression in all treatment groups than in the control, with the highest levels in the niclosamide and combination groups (Figure 5B). These results indicated that niclosamide substantially contributed to DNA damage accumulation in RCC cells.

To further investigate the kinetics of DNA damage induction and repair, we performed time-course immunofluorescence staining of γ-H2AX and 53BP1 after transient DNA damage induction with a 30 min H_2_O_2_ pulse, followed by treatment with DMSO, sunitinib, niclosamide, or the combination. γ-H2AX and 53BP1 foci were quantified as established markers of DNA double-strand break signaling and repair resolution [15,16,17].

No detectable γ-H2AX or 53BP1 foci were observed 0, 1, or 2 h after H_2_O_2_ exposure. Therefore, subsequent analyses focused on 8 and 24 h time points. Niclosamide and combination treatments resulted in abundant γ-H2AX and 53BP1 foci at both time points, with no significant decline from 8 to 24 h. In contrast, H_2_O_2_-treated control cells showed a marked reduction in foci by 24 h, consistent with efficient DNA repair (Figure 5C and Supplementary Figure S6A and B).

Quantification confirmed that the proportions of γ-H2AX- and 53BP1-positive cells were significantly higher in the niclosamide and combination groups than in the H_2_O_2_ control group at both 8 and 24 h (Figure 5D). Notably, γ-H2AX marks the chromatin regions flanking DNA double-strand breaks (DSBs), whereas 53BP1 is a DNA repair factor recruited to these sites to facilitate non-homologous end joining. The sustained co-localization of γ-H2AX and 53BP1 foci in niclosamide- and combination-treated cells suggests persistent recruitment of repair factors to DSB sites without timely resolution. This pattern is consistent with the delayed resolution of DNA lesions and may indicate prolonged DDR. These findings suggest that niclosamide induces DNA damage and results in persistent γ-H2AX- and 53BP1 foci. Such persistence is in line with a potential delay or impairment in DNA repair and could also contribute to the observed G0/G1 arrest and apoptosis.

### 2.6. Niclosamide Down-Regulates Key Regulators of the DNA Repair and the Cell Cycle Pathways

To identify the critical genes regulated by niclosamide, we integrated DEGs from our RNA-seq data with transcriptomic data from TCGA-KIRC dataset. KEGG pathway enrichment analysis (Supplementary Table S1) revealed that 44 downregulated genes were significantly enriched in pathways related to DNA repair and cell cycle regulation following niclosamide treatment.

**Figure 6:**
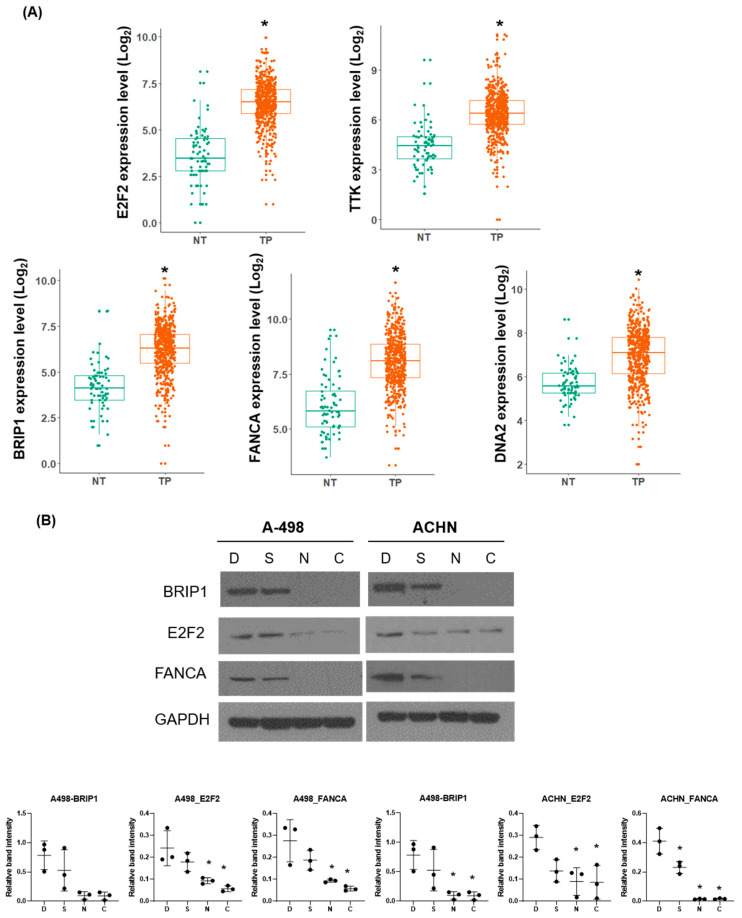
Correlation between experimental and clinical expression of niclosamide-regulated genes. (**A**) BRIP1, E2F2, FANCA, DNA2, and TTK mRNA expression levels in normal tissue (NT) and primary tumor tissue (TP) from TCGA-KIRC dataset. (**B**) A-498 and ACHN cells were treated with 2.5 µM sunitinib, 1 µM niclosamide, or their combination, for 48 h. Protein expression of BRIP, E2F2, and FANCA was detected by Western blot; GAPDH was a loading control. Representative blots from n = 3 independent experiments are shown. Densitometric quantification of γ-H2AX relative to GAPDH is presented as mean ± SD (n = 3). Statistical significance was determined by one-way ANOVA with post hoc tests. (* *p* < 0.05 compared to control). D, DMSO; S, sunitinib; N, niclosamide; C, combination.

To determine the genes with clinical importance and therapeutic relevance, we screened their expression levels in TP (primary tumor tissue) compared with NT (normal tissue) using TCGA-KIRC database (Log FC > 2.0, FDR < 0.05). This filtering step identified five genes, E2F2, TTK, BRIP1 (also known as FANCJ), FANCA, and DNA2, that were significantly upregulated in TP (Figure 6A). In contrast, the other 39 genes did not show significant differential expression between the TP and NT groups.

Next, we validated the effects of niclosamide and the combination treatment on the expression of these five genes. In qRT-PCR, the relative BRIP1, E2F2, and FANCA mRNA expression levels in both A-498 and ACHN cells were significantly decreased by niclosamide or combination treatment than by DMSO or sunitinib treatment alone (Supplementary Figure S7A and B). We confirmed these findings at the protein level by Western blot analysis. Consistent with the qRT-PCR results, BRIP1, E2F2, and FANCA levels were markedly decreased in RCC cells treated with niclosamide and the combination therapy (Figure 6B).

## 3. Discussion

Development of resistance to TKIs, such as sunitinib, remains a major clinical challenge in the treatment of RCC. Owing to the limited efficacy of TKIs and biological complexity of RCC, combination strategies targeting distinct molecular pathways have been explored to overcome drug resistance and improve treatment outcomes [6]. To the best of our knowledge, this is the first study to demonstrate that the combination of sunitinib and niclosamide exerts a synergistic anticancer effect in RCC. Mechanistically,, niclosamide modulates the DNA damage response (DDR) and cell-cycle regulation, thereby reinforcing tumor vulnerability to sunitinib. These findings support drug repurposing as a viable approach for enhancing therapeutic efficacy in RCC.

Niclosamide has been reported to exert antitumor effects in several epithelial cancers by modulating diverse signaling pathways, such as Wnt/β-catenin, mTOR, and STAT3 [9]. Similar pleiotropic effects have also been observed in RCC models [12,13]. Consistent with these reports, our data confirmed that niclosamide induced apoptosis and suppressed cell migration and invasion. However, as a monotherapy, niclosamide alone appears to be insufficient for complete tumor control, prompting the investigation of its use in combination regimens. In our models, the niclosamide-sunitinib combination produced a synergistic effect, as evidenced by the synergy score and markedly enhanced tumor suppression without notable toxicity.

Sunitinib is well recognized for its anti-angiogenic activity through inhibition of VEGFR and PDGFR signaling. Our findings suggest that niclosamide may also contribute modestly to anti-angiogenic effects, as evidenced by a reduction in CD31 expression. However, niclosamide appeared to exert effects beyond angiogenic inhibition. Consistent with its pleiotropic activity, our transcriptomic profiling revealed a mechanistic pattern in RCC characterized by downregulation of genes involved in cell cycle regulation and DNA repair, leading to impaired DNA damage response (DDR) and apoptosis [18]. Similar DDR-related effects have been reported in other cancers [9].

Although a few up-regulated genes were also identified, KEGG pathway analysis indicated enrichment in protein processing in the endoplasmic reticulum and peroxisome pathways, suggesting activation of ER- and oxidative-stress responses as secondary effects of niclosamide treatment. Functional validation confirmed that niclosamide induces DNA damage, as evidenced by sustained γ-H2AX and 53BP1 foci, and G0/G1 arrest, collectively suggesting impaired DNA repair and checkpoint disruption leading to apoptosis. These findings are consistent with those of previous studies in hepatocellular carcinoma, thyroid cancer, and pancreatic cancer models [19,20,21], and our previous study in prostate cancer showing niclosamide-mediated suppression of tumor growth via DDR modulation [11]. Together, these complementary mechanisms, involving anti-angiogenic activity of sunitinib and suppression of distinct molecular pathways by niclosamide, likely account for the additive and synergistic efficacy observed in RCC. This combination was rationally designed based on their distinct targeting mechanisms, allowing niclosamide to complement pathways not effectively inhibited by sunitinib.

G1 phase plays a pivotal role in determining cell fate, during which cells either repair DNA damage or commit apoptosis [22]. By disrupting this checkpoint, niclosamide may compromise the DDR pathway, which has been implicated in RCC progression and recurrence [23,24]. Targeting both DDR and cell cycle regulation, as achieved by niclosamide in combination with sunitinib, may therefore represent a promising strategy to enhance efficacy and overcome resistance in RCC. This is consistent with previous reports showing that other agents inducing cell cycle arrest can effectively suppress RCC progression [25,26,27,28]. Although immune checkpoint inhibitor (ICI)-based combinations with TKIs have become the standard of care for metastatic RCC, resistance and treatment intolerance remain major challenges [29]. The present findings provide a mechanistic rationale for exploring niclosamide-based regimens as potential strategies to overcome these limitations.

Furthermore, we identified BRIP1, FANCA, and E2F2 as clinically relevant targets modulated by niclosamide through integrated analysis of TCGA-KIRC data. In the FA pathway, FANCA and BRIP1 play key roles in DNA damage repair. FANCA participates in the repair of DNA interstrand crosslinks and double-stranded breaks (DSBs) via the canonical FA pathway [30]. FANCA deficiency has been linked to increased tumorigenicity and poor prognosis of leukemia [31,32]. BRIP1 contributes to genomic stability by facilitating the repair of DSBs and crosslinks [33]. Moreover, overexpression of BRIP1 is a prognostic biomarker for breast and gastric cancers [34,35].

In contrast, E2F2 functions as a key regulator of G1/S phase transition and initiates DNA replication [36]. Elevated E2F2 expression promotes proliferation and confers gemcitabine resistance in pancreatic cancer [37] and is also elevated in patients with RCC, suggesting its potential utility as a diagnostic biomarker [38]. Collectively, these findings suggest that BRIP1, FANCA, and E2F2 may be important mediators of the effects of niclosamide and potential therapeutic targets in RCC.

This study has several limitations. First, the in vivo validation was performed using a single RCC cell line (ACHN) with a small cohort size (n = 5 per group), which may not fully capture the heterogeneity of RCC. Future studies using additional clear cell RCC models, such as A-498 and Caki-1, will be essential to further validate these findings and confirm their generalizability. However, the synergistic anti-proliferative effects were consistently reproduced across three RCC cell lines (ACHN, A-498, and Caki-1) in vitro (Figure 2), and the key mechanistic findings related to DDR and cell cycle regulation were validated in A-498 and ACHN (Figure 5 and Supplementary Figure S7). Second, while a comet assay was not performed, time-course analysis of γ-H2AX and 53BP1 foci provided indirect evidence of impaired DNA damage repair. Third, although BRIP1, FANCA, and E2F2 were identified as potential mediators of the effects of niclosamide, their roles have not yet been directly validated through knockdown or rescue experiments. Finally, niclosamide has limited oral bioavailability in humans; therefore, further studies should include comprehensive pharmacokinetic and pharmacodynamics analyses, including plasma and tumor concentration measurements, as well as formulation optimization and dose-finding experiments, to ensure translational feasibility. In this regard, recent formulation advances, such as salt, solid dispersion, and nanoparticle-based preparations, are being actively explored to overcome these limitations and improve systemic exposure [39]. Despite these limitations, our results provide strong preclinical evidence that niclosamide is a promising partner for combination therapy with sunitinib for RCC. Furthermore, the mechanistic concept demonstrated in this study, targeting both angiogenic and DDR/cell cycle pathways, provides proof-of-concept for DDR-targeted combination strategies. As the therapeutic paradigm of RCC continues to shift toward newer multi-kinase inhibitors, further validation using contemporary TKIs such as cabozantinib or lenvatinib will be essential to confirm whether this mechanism extends beyond sunitinib. 

## 4. Materials and Methods

### 4.1. Reagents and Cell Cultures

Sunitinib malate and niclosamide were purchased from Sigma-Aldrich (St. Louis, MO, USA), dissolved in dimethyl sulfoxide (DMSO), and stored at −20 °C.

Human RCC cell lines A-498, ACHN, and Caki-1 were purchased from the Korean Cell Line Bank (Seoul, Republic of Korea). A-498 and ACHN cells were cultured in Dulbecco’s modified Eagle’s medium (Gibco, Thermo Fisher Scientific, Inc., Waltham, MA, USA) supplemented with 10% fetal bovine serum (FBS; Gibco) and 100 U/mL penicillin–streptomycin (Gibco). Caki-1 cells were cultured in RPMI 1640 medium (Gibco) supplemented with 10% FBS and 100 U/mL penicillin–streptomycin. All cells were incubated at 37 °C in a humidified atmosphere containing 5% CO_2_.

A-498 and Caki-1 are widely used clear cell RCC (ccRCC) models derived from primary and metastatic tumors, respectively. ACHN, classified as papillary RCC (pRCC), was included because it was derived from a metastatic pleural effusion and is commonly used as a model for metastatic and drug-resistant RCC. This cell-line panel was selected to represent the histologic and molecular diversity of RCC.

### 4.2. Cell Viability Assay

Sunitinib has been approved as an alternative therapy for advanced RCC. Although its apoptotic effects on RCC cells have been previously demonstrated, the concentration required to induce apoptosis was markedly higher than pharmaceutically relevant doses [40]. Cells were seeded in 96-wells plates and treated with various concentrations of sunitinib, niclosamide, or their combination, based on the pretreatment methods reported in previous studies [12,40]. Cell viability was assessed using EZ-CYTOX reagent (Daeil Lab Service Co. Ltd., Seoul, Republic of Korea) according to the manufacturer’s instructions. The absorbance was measured at 450 nm using a microplate reader.

### 4.3. Apoptosis Assay

After 48 h of drug treatment, the cells were harvested, washed three times with phosphate-buffered saline (PBS), and stained with Annexin V and propidium iodide (PI) using a commercial kit (Annexin-FITC/PI staining kit, BD Biosciences, San Jose, CA, USA). The apoptotic cells were analyzed by flow cytometry (FACS Canto II system, BD Biosciences).

### 4.4. Wound Healing Assay and Invasion Assay

For the wound healing assay, the cells were seeded in six-well plates. After incubating the plates for 24 h, a wound was made using a 200 µL pipette tip. The cells were then treated with DMSO or niclosamide (0.5–5 µM). The wound area was observed under a light microscope (Carl Zeiss, Oberkochen, Germany). The assay was performed in three independent biological repeats (n = 3). Representative images are shown.

Cell invasion assays were conducted using 24-well Transwell chambers with an 8-μm pore polycarbonate membrane insert (Corning Inc., Corning, NY, USA) coated with Matrigel (Corning). Cells in serum-free medium were seeded in the upper chamber, and medium with DMSO or 0.5, 1, 5 µM niclosamide was added to the lower chamber, which also contained 10% FBS as a chemoattractant. After 48 h, the cells that had invaded to the bottom side of the membrane were fixed, stained with 0.1% crystal violet solution (Sigma-Aldrich), and observed under a light microscope (Carl Zeiss).

### 4.5. Drug Interaction and Synergy Analysis

To evaluate drug synergy, the cells were treated with single or combined agents at various concentrations. Cell viability was measured, and synergy scores were calculated using the Highest Single Agent (HAS) reference model in the SynergyFinder 2.0 (https://synergyfinder.fimm.fi/, accessed on 1 November 2022) [15]. The overall level of synergy or antagonism was summarized over the full dose–response matrix using the delta score, which quantifies the combination effect. The HAS synergy score was visualized as an inhibition heatmap and a synergistic landscape (2D and 3D).

### 4.6. In Vivo Study

All animal experiments were approved by the Institutional Animal Care and Use Committee of the Catholic University of Korea (CUMC-2018-0313-01) and were conducted during the approved study period (December 2018–July 2020). Male BALB/c nude mice (5 weeks old, body weight, 20–25 g) were obtained from Orient Bio (Gyeonggi-do, Republic of Korea). The mice were injected subcutaneously in the flank with 1 × 107 ACHN cells in 100 µL PBS with 100 µL Matrigel (Corning), as previously described in RCC models [39]. When the tumor volumes reached approximately 250 mm^3^, the mice were randomly assigned to four groups (each containing five mice) based on similar average tumor size: vehicle, sunitinib, niclosamide, or a combination.

The animals were treated with vehicle, sunitinib (20 mg/kg), niclosamide (20 mg/kg), or the combination, administered by daily oral gavage for four weeks. The dose was selected based on previous studies that demonstrate in vivo efficacy and tolerability in xenograft models [12,15]. The mice were monitored daily for signs of distress, including normal posture, hypoactivity, and feeding behavior. Body weight and tumor size were measured every three days, and tumor volume was calculated as width2 × length × 0.5. No animals showed signs of overt toxicity, and body weights remained within 90–110% baseline during the treatment period. After four weeks of treatment, mice were euthanized by gradual-fill CO_2_ inhalation at a displacement rate of ~20–30% of the chamber volume per minute, followed by cervical dislocation to ensure death, in accordance with AVMA Guidelines (2020) and institutional SOPs. Death was verified by the absence of heartbeat and reflexes. Tumors were then harvested for further analysis.

### 4.7. Histological Analyses and Immunohistochemistry

The excised tumor tissues were fixed, paraffin-embedded, cut into 4 µM sections, and stained with hematoxylin and eosin. The paraffin sections were immunostained with rabbit Ki-67 (1:200; Abcam, Cambridge, UK) and cleaved caspase-3 (1:50; Cell Signaling Technology, Danvers, MA, USA) primary antibodies. Species-matched horseradish peroxidase (HRP)-conjugated secondary antibodies were used to detect the primary antibodies. The color reaction of HRP-conjugated antibodies was performed using a DAB kit (Vector Laboratories Inc., Burlingame, CA, USA). Tissue sections were examined under a light microscope (Carl Zeiss).

### 4.8. mRNA Sequencing and Pathway Analysis

Total RNA was extracted from ACHN cells treated with 2.5 µM sunitinib and 1 µM niclosamide alone or in combination. Libraries were prepared from total RNA using the NEBNext Ultra Directional RNA-Seq Kit (New England BioLabs, Inc., Rowley, MA, USA). High-throughput sequencing was performed as paired-end 100 pb sequencing using HiSeq 2500 (Illumina, Inc., San Diego, CA, USA). The mRNA sequencing (mRNA-seq) reads were mapped using TopHat software, version 2.1.1 (Johns Hopkins University, Baltimore, MD, USA) to obtain an alignment file [41,42]. Differential expression analysis was performed using the ExDEGA software, version 1.6.5 E-Biogen Inc., Seoul, Republic of Korea) to identify genes that were significantly altered in each treatment group (sunitinib, niclosamide, or combination) compared with vehicle treatment controls. Genes with a fold change > 1.5 and *p* < 0.05 were considered differentially expressed. Venn diagram analysis was then conducted to identify genes commonly dysregulated in niclosamide and combination treatments but not in sunitinib alone, representing niclosamide-driven transcriptional changes. Kyoto Encyclopedia of Genes and Genomes (KEGG) pathway analysis of DEGs was performed using DAVID bioinformatics tool (https://david.ncifcrf.gov/, accessed on 1 August 2022). The RNA-seq data generated in this study have been deposited in the NCBI Gene Expression Omnibus (GEO) under accession number GSE306717.

### 4.9. Western Blot Analysis

Cell lysates were prepared with RIPA lysis buffer (Cell Signaling Technology). Proteins were separated by sodium dodecyl sulfate–polyacrylamide gel electrophoresis and transferred onto nitrocellulose membranes. The membranes were blocked and subsequently incubated overnight at 4 °C with specific primary antibodies against BRIP1 (Cell Signaling Technology), E2F2 (Santa Cruz Biotechnology, Inc., Dallas, TX, USA), FANCA (Cell Signaling Technology), γH2AX (Ser139; Cell Signaling Technology), Cyclin A (Santa Cruz Biotechnology, Inc.), Cyclin B (Santa Cruz Biotechnology, Inc.), CDK1 (Santa Cruz Biotechnology, Inc.), β-actin (Santa Cruz Biotechnology, Inc.), and GAPDH (Santa Cruz Biotechnology, Inc.). The membranes were incubated with the appropriate secondary antibodies (GenDEPOT, Barker, TX, USA) and the protein bands were visualized using an ECL kit (DoGenBio, Seoul, Republic of Korea). All Western blot experiments were performed in three independent biological repeats (n = 3), and representative blots are shown. Band intensities were quantified using image J softe ware, version 1.53t (NIH, Bethesda, MD, USA https://imagej.net/, accessed on 1 May 2025) and normalized to GAPDH. Data from three independent experiments are presented as mean ± SD.

### 4.10. Cell Cycle Assay

The cells were treated with drugs, fixed in ice-cold 75% ethanol, washed twice with PBS, and incubated with 100 µg/mL RNase A and 50 µg/mL PI. Cell cycle distribution was analyzed using a flow cytometer (FACS Canto II system, BD Biosciences).

### 4.11. Immunocytochemistry

The cells were grown on cell culture slides (SPL Life Sciences, Cat. No. 30508, Pocheon-si, Gyeonggi-do, Republic of Korea) and subjected to a 30 min pulse treatment with H2O2 to induce DNA damage, followed by medium replacement and incubation with the indicated drugs. The cells were then fixed with 4% formaldehyde and permeabilized with 0.1% Triton X-100. The samples were further incubated with primary antibodies against γH2AX (Ser139; 1;200; rabbit; Cell Signaling Technology) and 53BP1 (1:150; mouse; Abcam), followed by washing with PBS. The samples were then incubated with secondary antibodies conjugated to FITC or Alexa 568. Nuclei were counterstained with DAPI and confocal images were acquired using an LSM510 Meta confocal laser scanning microscope (Carl Zeiss, Oberkochen, Germany).

The number of γH2AX- and 53BP1-positive foci per nucleus was manually counted using merged images. At least 50 nuclei per condition were analyzed for each of three independent biological replicates.

Co-localization was defined as the presence of overlapping γH2AX (green) and 53BP1 (red) foci within the same nucleus in merged images. The proportion of co-localization was calculated as the number of nuclei containing at least one co-localized focus. This value was divided by the total number of nuclei analyzed per field.

### 4.12. TCGA Dataset

We used the TCGAbiolinks package, version 2.26.3 (Bioconductor, https://bioconductor.org/packages/TCGAbiolinks, accessed on 1 August 2023) to download RNA-seq and clinical data from the Genomic Data Commons (GDC) data portal on January 20, 2020. These databases (kidney renal clear cell carcinoma; KIRC), were generated using the Illumina HiSeq RNASeq platform and included mRNA sequencing data from 533 clear cell RCC (ccRCC) tissues (primary solid tumor; TP) and 72 adjacent non-tumorous renal tissue (normal tissue; NT) samples. All data were normalized and processed using the TCGAbiolinks pipeline. The TCGAbiolinks principle of differentially expressed gene (DEG) analysis is as follows [43]. The cutoff criteria were FDR < 0.01 and |Log2FC| > 2.0.

### 4.13. Quantitative Reverse Transcription-PCR (qRT-PCR)

The RNA from the cells was extracted using TRIzol^®^ reagent (Invitrogen Life Technologies; Thermo Fisher Scientific, Inc., Waltham, MA, USA), following the manufacturer’s protocol. Total RNA (1 µg) was reverse-transcribed to cDNA using the PrimeScriptTM RT reagent kit (Takara Bio Inc., Shiga, Japan), and quantitative PCR was carried out using the TB Green™ Premix Ex Taq™ II (Takara Bio Inc.). The primer pairs used are listed in Supplementary Table S2. Relative expression was normalized to that of GAPDH.

### 4.14. Statistical Analysis

All experimental data are expressed as mean ± standard deviation (SD), except for in vivo tumor growth data, which are expressed as mean ± standard error of the mean (SEM).

Statistical analyses were performed using GraphPad Prism software (GraphPad Software; Version 10.0; San Diego, CA, USA). For comparisons between the two groups, an unpaired two-tailed Student’s *t*-test was used. For comparison among more than two groups, a one-way analysis of variance (ANOVA) followed by Tukey’s post hoc test was used. For longitudinal tumor growth data in the xenograft model, two-way repeated-measures ANOVA with Geisser Greenhouse correction was used to assess the effects of treatment and time, followed by Tukey’s multiple comparison test.

For analyzing the proportion of γ-H2AX- and 53BP1-positive cells, as well as co-localization, two-way ANOVA was used to evaluate the effects of treatment and time, followed by Tukey’s multiple comparisons test. A *p*-value < 0.05 was considered statistically significant.

## 5. Conclusions

In this study, we demonstrated that niclosamide synergizes with sunitinib to exert potent anticancer effects in RCC. Niclosamide enhanced the efficacy of sunitinib by inducing DNA damage, impairing repair processes, and causing cell cycle arrest through the downregulation of BRIP1, FANCA, and E2F2. These findings provide proof-of-concept evidence for DDR-targeted combination strategies as a potential therapeutic approach for RCC. 

## 6. Patents

This work has led to the registration of a patent: Pharmaceutical composition comprising niclosamide and sunitinib for preventing or treating kidney cancer. Korean Patent No. 10-2595271, filed on 12 October 2021 and registered on 23 October 2023. Patentee: Catholic University of Korea Industry–Academic Cooperation Foundation. Inventors: Yong Hyun Park, Ae Ryang Jung, Ga Eun Kim, Mee Young Kim, and Na Yoon Kim.

## Data Availability

The RNA-seq data generated in this study have been deposited in the NCBI Gene Expression Omnibus (GEO) under accession number GSE306717 (https://www.ncbi.nlm.nih.gov/geo/query/acc.cgi?acc=GSE306717 (accessed on 28 August 2025). The dataset is currently under embargo and will be publicly released on 28 August 2026. For peer review, the data can be accessed via a private reviewer link. Public datasets used in this study are available from the GEO (https://www.ncbi.nlm.nih.gov/geo) and the TCGA Research Network TCGA Research Network (https://portal.gdc.cancer.gov/, accessed on 1 June 2022).

## Supplementary Materials

The following supporting information can be downloaded at: https://www.mdpi.com/article/10.3390/ijms262210922/s1.

## Abbreviations

The following abbreviations are used in this manuscript:

RCC	Renal cell carcinoma
TKIs	Tyrosine kinase inhibitors
VEGFR	Vascular endothelial growth factor receptor
PDGFR	Platelet-derived growth factor receptor
FA	Fanconi anemia
DSBs	DNA double-strand breaks
DDR	DNA damage response
DEG	Differentially expressed gene
SD	Standard deviation
SEM	standard error of the mean
ANOVA	A one-way analysis of variance
NT	Normal Tissue
TP	Primary Tumor Tissue
