# Supplementary material for: Novel Therapeutic Strategy for Renal Cell Carcinoma: Niclosamide Enhances Sunitinib Efficacy via DNA Repair and Cell Cycle Pathways
Source: Int J Mol Sci. 2025 Nov 11;26(22):10922. doi: 10.3390/ijms262210922 (PMC12653009; doi:10.3390/ijms262210922)
Supplement: Supplementary file 1 [file ijms-26-10922-s001.zip › ijms-3909176-supplementary.pdf]

Supplementary Table S1. Enriched KEGG pathways identified by gene set enrichment analysis.

Term	Count	*p*-value	Genes
**hsa04110:Cell cycle**	**24**	**1.26E-10**	**CDC7, E2F2, CDK1, YWHAZ, RBL1, SKP2, YWHAB, TTK, CHEK1, CDK6, CHEK2, MCM2, SMC3, WEE1, CDC25B, CCNE2, YWHAH, YWHAQ, PCNA, ORC5, ORC6, ABL1, SMC1A, TFDP1**
**hsa03030:DNA replication**	**12**	**4.36E-08**	**POLD3, RPA1, PRIM1, DNA2, RFC3, RFC4, POLE, PRIM2, PCNA, POLA1, MCM2, RNASEH2A**
**hsa03460:Fanconi anemia pathway**	**11**	**2.17E-05**	**RPA1, RAD51C, BLM, FANCD2, FANCI, FAAP24, BRIP1, FANCA, RMI1, UBE2T, BRCA1**
**hsa03430:Mismatch repair**	**6**	**0.001378**	**POLD3, RPA1, RFC3, RFC4, MSH2, PCNA**
hsa03040:Spliceosome	14	0.001379	SRSF10, TRA2B, LSM6, RBMX, HNRNPU, HNRNPA3, HNRNPK, HSPA2, SRSF7, DHX15, SNRNP40, SNRPE, HSPA8, RBM17
hsa05203:Viral carcinogenesis	18	0.001708	CDK1, YWHAZ, ACTN4, RBL1, SKP2, YWHAB, ACTN1, CHEK1, CDK6, SRF, PKM, CCNE2, CDC42, NRAS, CASP3, YWHAH,

			HNRNPK, YWHAQ
hsa05130:Pathogenic Escherichia coli infection	8	0.002712	ACTB, CDC42, EZR, TUBB6, ARPC4, ABL1, TUBA1C, CTNNB1
hsa00240:Pyrimidine metabolism	11	0.004404	POLD3, PRIM1, TYMS, UMPS, POLE, PRIM2, DHODH, DCK, POLA1, POLR2D, DUT
hsa03013:RNA transport	15	0.005061	UPF1, NUP85, NUP155, PNN, NDC1, SUMO3, EIF4G2, NUP62, EIF1AX, NUP50, RPP30, THOC7, NUP107, TGS1, GEMIN5
hsa05100:Bacterial invasion of epithelial cells	9	0.008507	ACTB, CDC42, CAV1, ARHGEF26, BCAR1, ARPC4, SEPT11, CTNNB1, VCL
hsa04810:Regulation of actin cytoskeleton	16	0.012206	ACTB, FGFR4, PDGFB, ACTN4, LIMK1, BCAR1, IQGAP3, ACTN1,ARPC4, VCL, CDC42, NRAS, EZR, CFL1, PIP4K2A, F2R
hsa04115:p53 signaling pathway	8	0.012276	CCNE2, CDK1, CASP3, SERPINE1, CHEK1, CDK6, CHEK2, THBS1
hsa04520:Adherens junction	8	0.016555	ACTB, PTPRJ, CDC42, ACTN4, ACTN1, PTPN1, CTNNB1, VCL
hsa03440:Homologous recombination	5	0.021947	POLD3, RPA1, RAD51C, XRCC2, BLM
hsa04510:Focal adhesion	15	0.022617	ACTB, CAV1, PDGFB, ACTN4, BCAR1, ACTN1, CAPN2, VASP, VCL, CTNNB1,

			CDC42, COL6A3, LAMC1, ZYX, THBS1
hsa04114:Oocyte meiosis	10	0.023104	CCNE2, CDK1, YWHAZ, YWHAH, PPP2R5D, YWHAB, YWHAQ, SMC1A, SMC3, CALM2
hsa04390:Hippo signaling pathway	12	0.026119	ACTB, YWHAZ, YWHAH, TP53BP2, SERPINE1, YWHAB, YWHAQ, TEAD2, LIMD1, GLI2, WWTR1, CTNNB1
hsa04670:Leukocyte transendothelial migration	10	0.028289	ACTB, CDC42, EZR, ACTN4, BCAR1, ACTN1, CLDN2, VASP, CTNNB1, VCL
hsa03420:Nucleotide excision repair	6	0.030341	POLD3, RPA1, RFC3, RFC4, POLE, PCNA
hsa04530:Tight junction	8	0.044133	ACTB, CDC42, ACTN4, CGN, ACTN1, CLDN2, AMOTL1, MYH9
hsa05161:Hepatitis B	11	0.045447	CCNE2, NRAS, E2F2, CASP3, YWHAZ, PCNA, YWHAB, YWHAQ, FADD, CDK6, NFATC2
hsa04360:Axon guidance	10	0.048634	CDC42, NRAS, NRP1, LIMK1, CFL1, DPYSL5, SEMA4D, NFATC2, ABL1, EPHB2
hsa04015:Rap1 signaling pathway	14	0.051695	ACTB, FGFR4, PDGFB, BCAR1, KITLG, APBB1IP, VASP, CTNNB1, CDC42, NRAS, RAPGEF5, THBS1, CALM2, F2R
hsa03050:Proteasome	5	0.081512	PSMC6, PSMA4, PSMA3, PSMD1, PSME3

hsa04151:PI3K-Akt signaling pathway	19	0.096590	SGK1, YWHAZ, FGFR4, PHLPP2, PDGFB, PPP2R5D, YWHAB, KITLG, CDK6, BRCA1, CCNE2, NRAS, YWHAH, COL6A3, YWHAQ, LAMC1, THBS1, PPP2R3C, F2R
hsa05222:Small cell lung cancer	7	0.098805	CCNE2, CKS1B, E2F2, CKS2, SKP2, CDK6, LAMC1

Supplementary Table S2. Primer sequences used for qRT-PCR.

Gene	Forward sequence (5′ to 3′)	Reverse sequence (5′ to 3′)
**BRIP1**	GGAAGAAGCAGGGAAAGCAG	GAGGCACTATTCTCTGATGACC
**E2F2**	GACTCGGTATGACACTTCGCTG	CGTTGGTGATGTCATAGATGCG
**TTK**	ATGATGATGGCAAACAACCC	CGCTTGACTGTAACGACCA
**FANCA**	GGTGGGTATTCTCTCAGCCG	CACAGGGTGACTGGTCTCCG
**DNA2**	GTGTTCTGTTCCAGTAGAGCCA	CCTAAAAGTTTCACTCAGGACAGC
**GAPDH**	GCTACAGCAACAGGGTGGTG	GGTCTACATGGCAACTGTGAGG
