# Supplementary material for: Novel Therapeutic Strategy for Renal Cell Carcinoma: Niclosamide Enhances Sunitinib Efficacy via DNA Repair and Cell Cycle Pathways
Source: Int J Mol Sci. 2025 Nov 11;26(22):10922. doi: 10.3390/ijms262210922 (PMC12653009; doi:10.3390/ijms262210922)
Supplement: Supplementary file 1 [file ijms-26-10922-s001.zip › supple figure legend .pdf]

Supplementary materials legend

Supplementary Figure S1.

A-498, ACHN, and Caki-1 cells were treated with the indicated concentrations of sunitinib for 24 and 48 h. Cell viability was measured using the WST assay. Data are mean ± SD (*$P < 0.05$ compared to control at the same time).

Supplementary Figure S2.

Representative Annexin V-FITC/PI flow-cytometry dot plots of A-498, ACHN, Caki-1 treated with niclosamide for 48 h. Columns: DMSO, 1 μM and 5 μM Niclosamide. Rows (cell lines): A-498 (top), ACHN (middle), Caki-1 (bottom). Quadrants are labeled as Q1 (Annexin V–/PI+), Q2 (Annexin V+/PI+; late apoptosis), Q3 (Annexin V+/PI–; early apoptosis), and Q4 (Annexin V–/PI–; viable), with the percentage of cells in each quandrant indicated. Representative of n = 3 independent experiments. Quantification is provided in Fig. 1B.

Supplementary Figure S3

(A) Dose-response curves showing percentage inhibition of cell viability by sunitinib and niclosamide as single agents in ACHN cells. **(B)** Dose-response matrix (inhibition) illustrating the combinatiorial effects of sunitinib and niclosamide at various concentarations. Each value represents the mean of three independent experiments. **(C)** Synergy landscapes (2D and 3D) based on the HSA model. Color gradients from green (negative δ-score) to red (positive δ-score high) indicated levels of synergy. Synergy scores > 0 indicate synergism; scores < 0 indicate antagonism.

Supplementary Figure S4

Representative Annexin V-FITC/PI flow-cytometry dot plots of A498, ACHN, Caki-1 treated with DMSO, sunitinib, niclosamide, or the combination for 48h. Columns: DMSO, Sunitinib, Niclosamide, Combination. Rows (cell lines): A-498 (top), ACHN (middle), Caki-1 (bottom). The percentage of cells in each quadrant is indicated. Quantification is provided in Fig. 2C.

Supplementary Figure S5

Representative cell-cycle DNA content histograms (PI;PE-A) corresponding to Fig. 5A. Cells were treated for 48 h with DMSO, sunitinib, niclosamide, or the combination. (A) A498; (B) ACHN. Representative of n = 3 independent experiments.

Supplementary Figure S6

(A) Nuclear γ-H2AX (green) and 53BP1 (red) foci in A498 and ACHN cells treated with DMSO for 8 h or 24 h. Nuclei were counterstained with DAPI (blue). **(B)** Representative ACHN cell images obtained under the same experimental conditions as in Figure 5C, showing γ-H2AX and 53BP1 foci following the indicated treatments. White arrows indicate co-localized foci in merged images. Scale bar = 20 μm.

Supplementary Figure S7

(A, B) A-498 and ACHN cells were treated with 2.5 μM sunitinib, 1 μM niclosamide, or their combination, for 48 h. mRNA expression levels of target genes were quantified by qRT-PCR and normalized to β-actin. Data are expressed as the mean ± SD (**P* < 0.05, ***P* < 0.01, as compared to control; $^{\#}P$< 0.05 compared to sunitinib).
