# Supplementary figures and images for: Novel Therapeutic Strategy for Renal Cell Carcinoma: Niclosamide Enhances Sunitinib Efficacy via DNA Repair and Cell Cycle Pathways
Source: Int J Mol Sci. 2025 Nov 11;26(22):10922. doi: 10.3390/ijms262210922 (PMC12653009; doi:10.3390/ijms262210922)

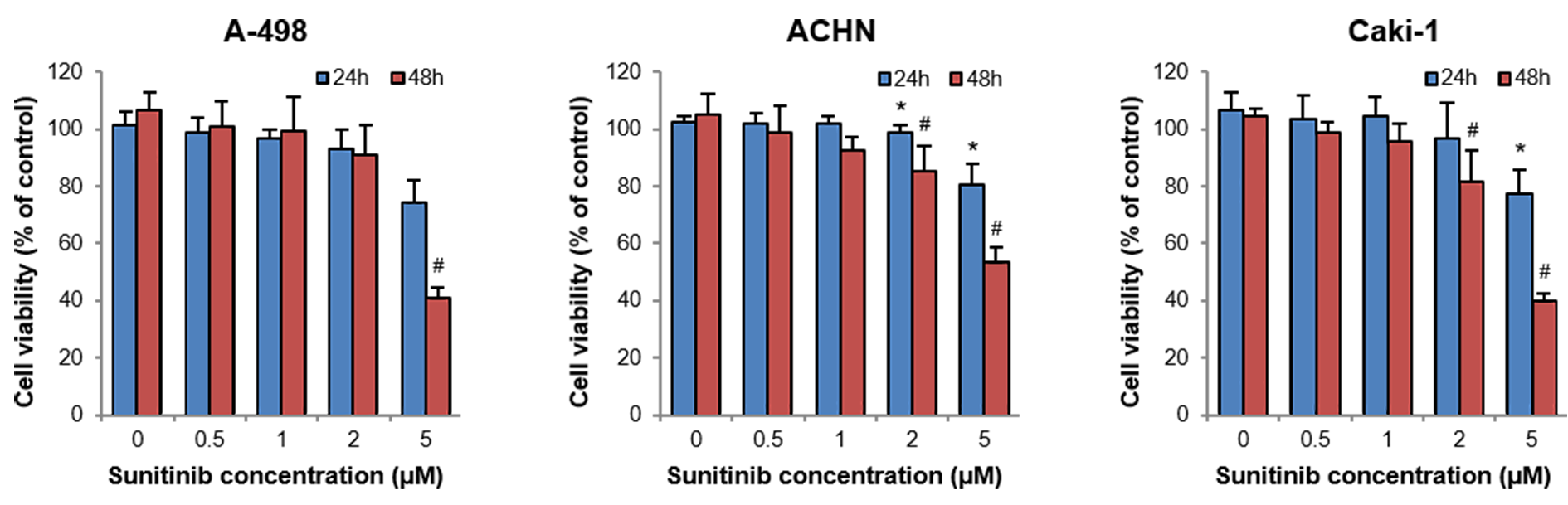

Supplement: Supplementary file 1 [file ijms-26-10922-s001.zip › Supple Figure S1.tif]

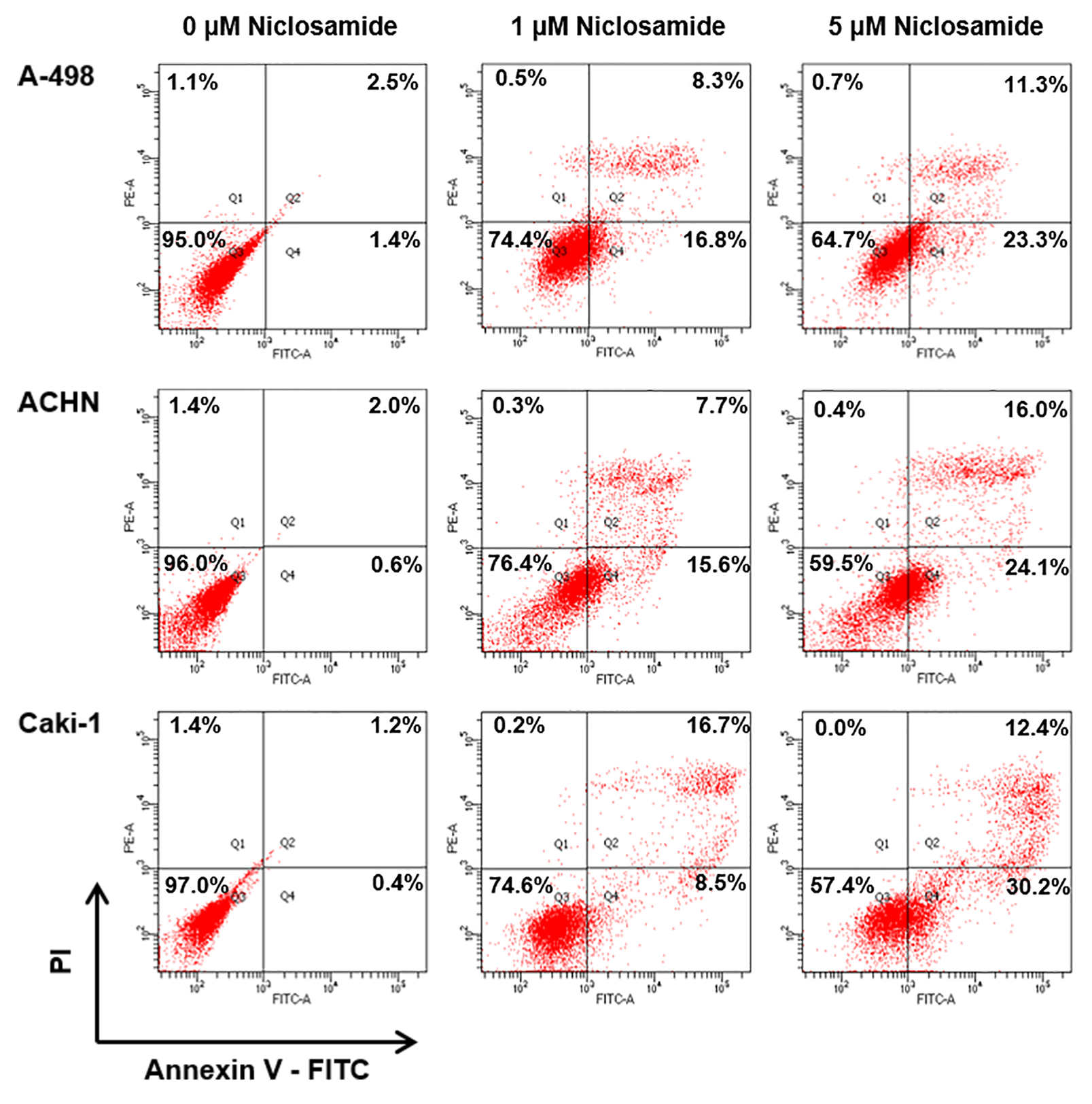

Supplement: Supplementary file 1 [file ijms-26-10922-s001.zip › Supple Figure S2.tif]

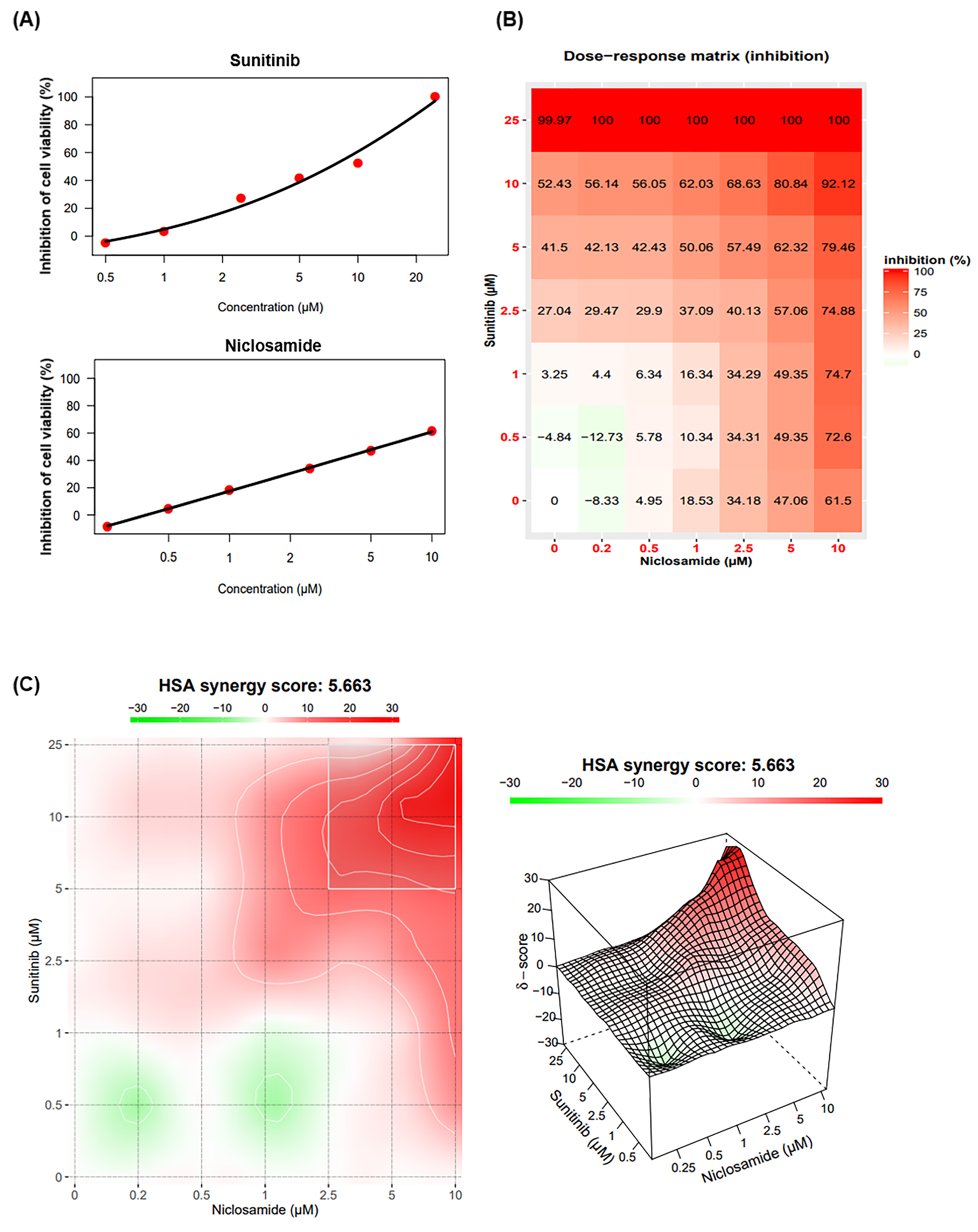

Supplement: Supplementary file 1 [file ijms-26-10922-s001.zip › Supple Figure S3.tif]

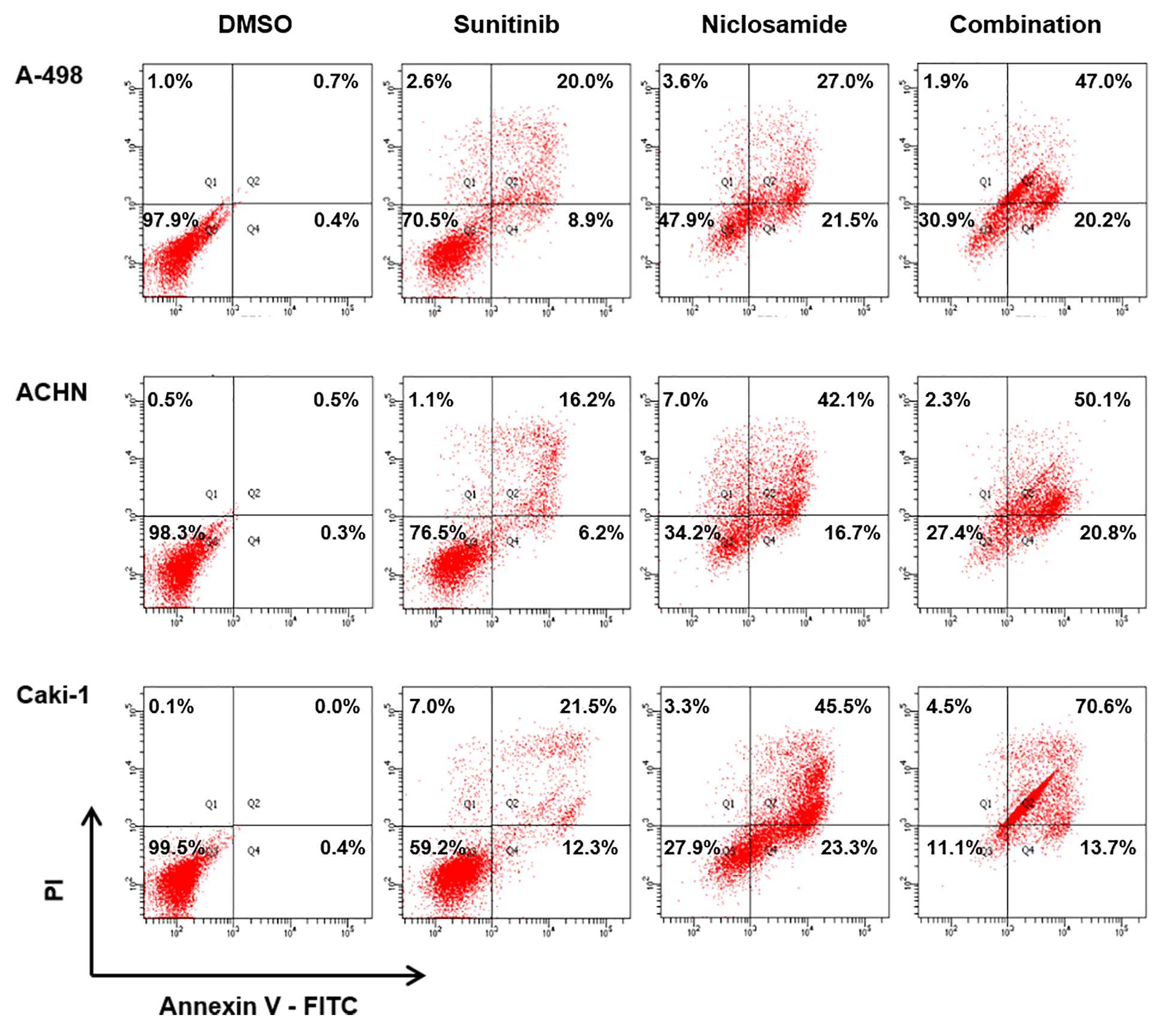

Supplement: Supplementary file 1 [file ijms-26-10922-s001.zip › Supple Figure S4.tif]

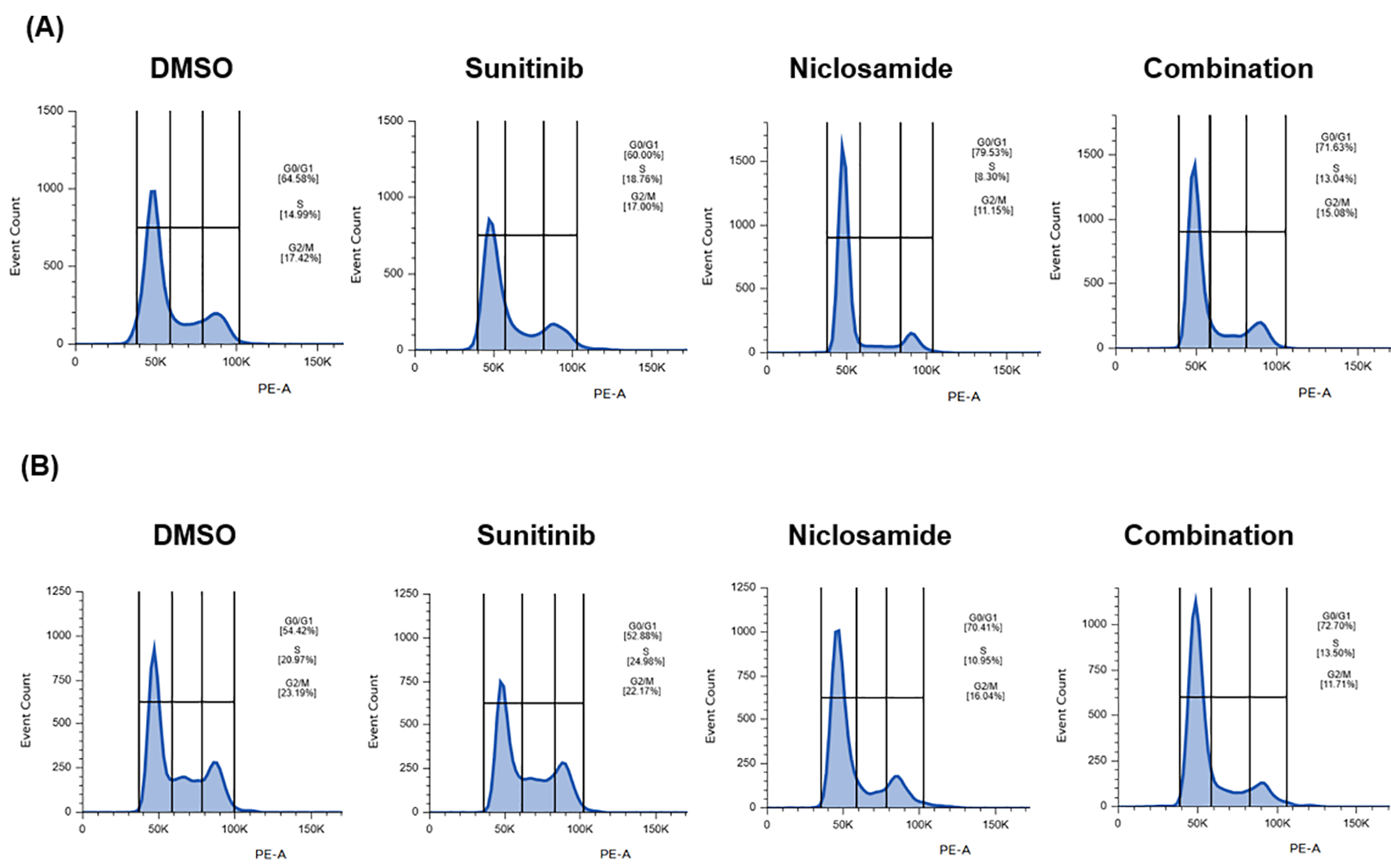

Supplement: Supplementary file 1 [file ijms-26-10922-s001.zip › Supple Figure S5.tif]

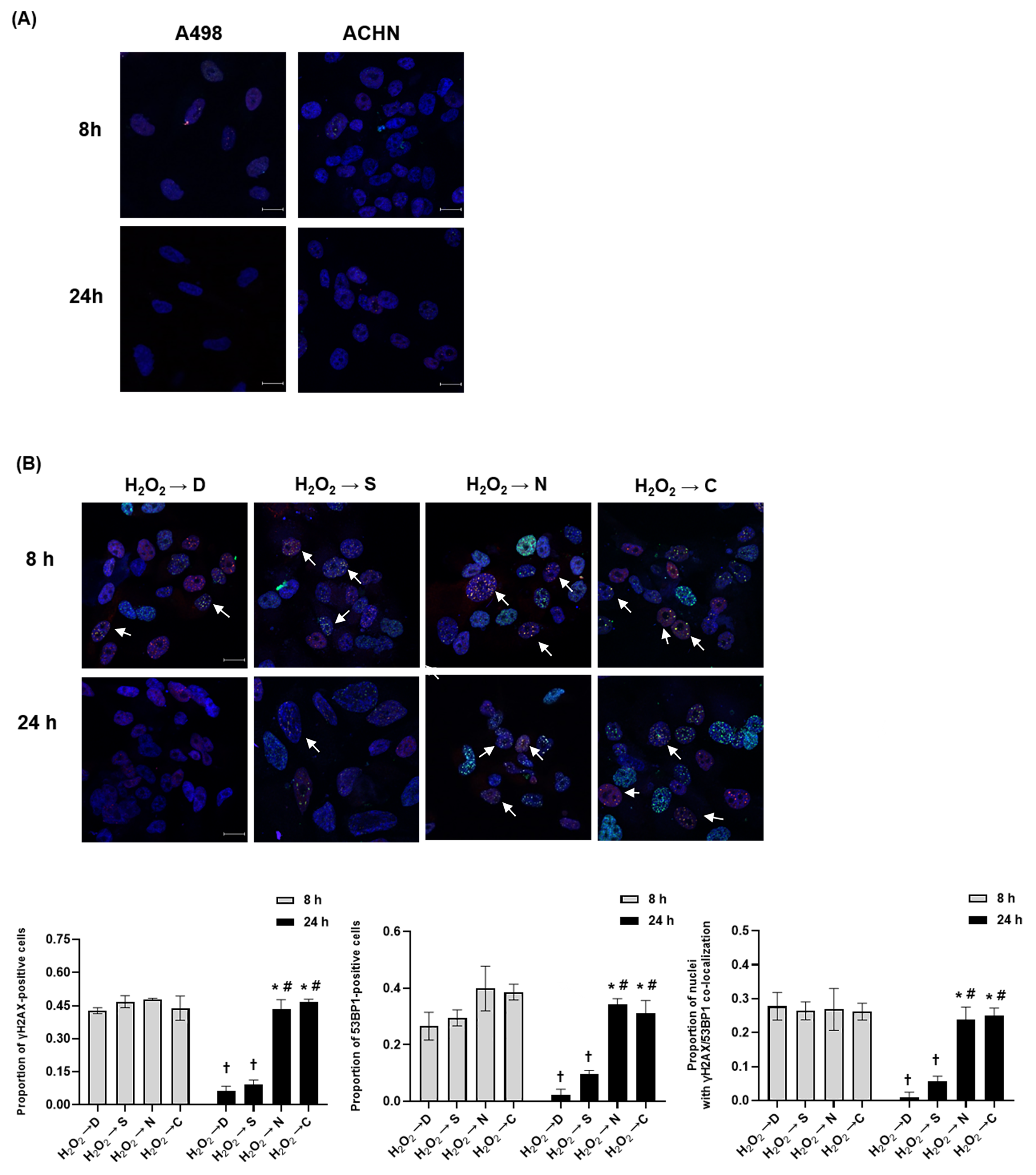

Supplement: Supplementary file 1 [file ijms-26-10922-s001.zip › Supple Figure S6.tif]

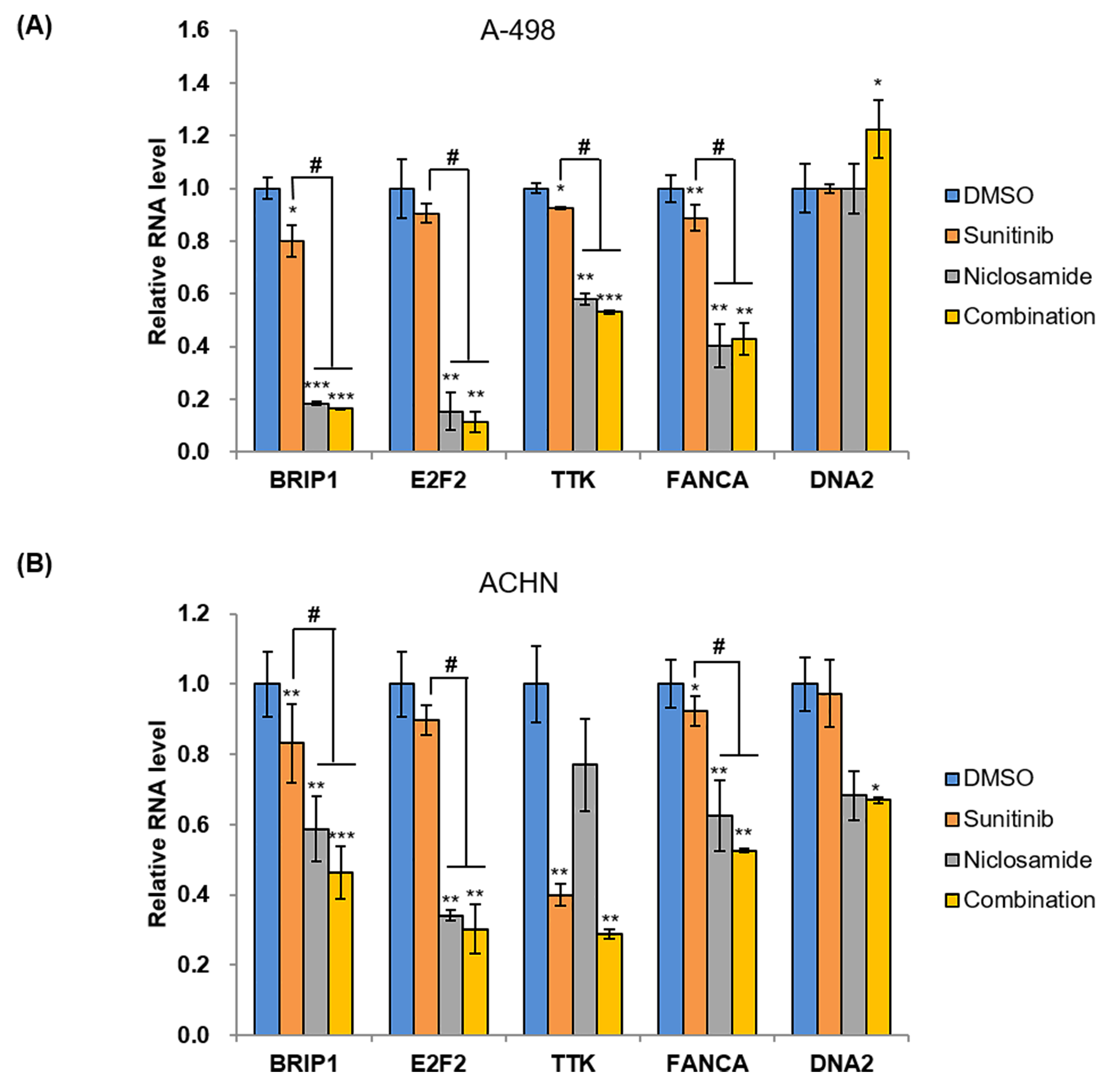

Supplement: Supplementary file 1 [file ijms-26-10922-s001.zip › Supple Figure S7.tif]
